# Strong modification of the transport level alignment in organic materials after optical excitation

**DOI:** 10.1038/s41467-019-09136-7

**Published:** 2019-04-01

**Authors:** Benjamin Stadtmüller, Sebastian Emmerich, Dominik Jungkenn, Norman Haag, Markus Rollinger, Steffen Eich, Mahalingam Maniraj, Martin Aeschlimann, Mirko Cinchetti, Stefan Mathias

**Affiliations:** 1 Department of Physics and Research Center OPTIMAS, University of Kaiserslautern, Erwin-Schrödinger-Straße 46, 67663 Kaiserslautern, Germany; 2 Graduate School of Excellence Materials Science in Mainz, Erwin-Schrödinger-Straße 46, 67663 Kaiserslautern, Germany; 3 Experimentelle Physik VI, Technische Universität Dortmund, 44221 Dortmund, Germany; 4 I. Physikalisches Institut, Georg-August-Universität Göttingen, Friedrich-Hund-Platz 1, 37077 Göttingen, Germany; 5 International Center for Advanced Studies of Energy Conversion (ICASEC), Georg-August-Universität Göttingen, 37077 Göttingen, Germany

## Abstract

Organic photovoltaic devices operate by absorbing light and generating current. These two processes are governed by the optical and transport properties of the organic semiconductor. Despite their common microscopic origin—the electronic structure—disclosing their dynamical interplay is far from trivial. Here we address this issue by time-resolved photoemission to directly investigate the correlation between the optical and transport response in organic materials. We reveal that optical generation of non-interacting excitons in a fullerene film results in a substantial redistribution of all transport levels (within 0.4 eV) of the non-excited molecules. As all observed dynamics evolve on identical timescales, we conclude that optical and transport properties are completely interlinked. This finding paves the way for developing novel concepts for transport level engineering on ultrafast time scales that could lead to novel functional optoelectronic devices.

## Abstract (web summary)

Correlating the optoelectronic properties in organic semiconductors is vital to realizing devices with high performance and new functionalities. Here, the authors report the role of optically excited charge transfer excitons on energy level alignment of the transport levels in organic thin films.

## Introduction

In the last decade, organic semiconductors have emerged as a novel class of functional materials with the potential to complement inorganic semiconductors for future electronic and spintronic applications^1–5^. The most intriguing property of organic materials is the chemical tunability of their structural and electronic properties. This opens a unique way to tune the spectral range of operation of organic optoelectronic devices, such as organic light emitting diodes (OLEDs) and organic photovoltaic cells, by designing the optical band gap^6–8^.

In general, organic optoelectronic devices rely intrinsically on the optical and electric transport properties of organic semiconductors that are commonly described invoking two quasi-particles: excitons and polarons. Understanding the still elusive correlation between excitons and polarons in organic semiconductors is of paramount importance. First of all, a potential modification of the transport levels induced by optical excitation could severely distort the energy level alignment that was specifically designed by chemical functionalization in the manufacturing process of the active organic material. This will have a significant impact on the efficiency of the entire photovoltaic device. Second, although organic semiconductors have already found their way into large scale technological implementation of OLEDs, exploiting the interplay between their optical and transport properties is expected to lead to a whole new range of device functionalities^9^.

From a fundamental point of view, the low dimensionality of organic semiconductors in combination with the weak van-der-Waals interactions in molecular crystals lead to a strongly reduced dielectric screening and to an enhanced Coulomb interaction between charge carriers^10–12^. These properties are responsible for pronounced many-body interactions, and thus influence quasiparticles such as excitons and polarons^13–17^. In other words, one expects a direct connection between the optical and transport properties in optoelectronic devices where optical excitations of charge carriers (excitons) and charge carrier transport (polarons) occur in direct vicinity and on similar timescales. Despite this intriguing link between excitonic and polaronic effects, the optical and transport properties of organic semiconductors have mainly been discussed separately from each other so far.

Here, we specifically address the correlation between optical and transport properties in organic thin films. We show clear spectroscopic signatures of transient changes of the linewidth of all occupied molecular transport levels upon optical excitation which can be directly linked to the characteristic timescales of the exciton decay of C_60_. Using micro-electrostatic simulations, we show that this transient broadening of all occupied molecular levels can be correlated to the dielectric screening of the molecular film due to the formation of localized and non-interacting excitons with different local charge distributions. Our combined experimental and theoretical approach hence shows that excitons of dominant charge transfer character act as charge defects in organic thin films that severely influence the transient energy positions of the transport levels of the entire molecular film.

## Results

### Optical and transport properties of organic thin films

**Fig. 1:**
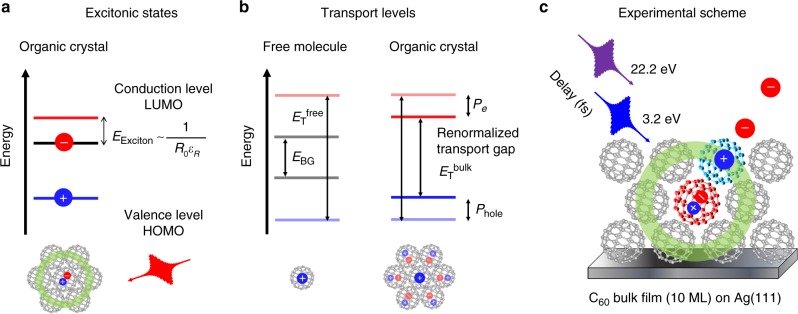
Optical and transport properties of organic crystals and overview of experimental approach. **a** Schematic illustration of the optically excited exciton level in a single particle energy level diagram. Optical excitation within the fundamental band gap from the highest occupied molecular orbital (HOMO) to the lowest unoccupied molecular orbital (LUMO) leads to the formation of an excitonic quasiparticle state within the HOMO–LUMO band gap with binding energy *E*_Exciton_. **b** Comparison of the energy level alignment of the (charged) transport levels (HOMO, LUMO) for free molecules and molecular crystals. The transport band gap $E_{\mathrm{T}}^{{\mathrm{bulk}}}$ of a molecular crystal is renormalized (reduced) with respect to one of the free molecules $E_{\mathrm{T}}^{{\mathrm{free}}}$ due to the long-range Coulomb interaction and the dielectric screening in organic thin films. **c** Schematic illustration of the time-resolved photoemission experiment. Optical excitation of a C_60_ molecular film results in the formation of localized Frenkel (as depicted for red molecule) or charge-transfer excitons. The quasiparticle dynamics of the optically excited C_60_ molecules as well as the transient modifications of the occupied transport levels of their molecular surrounding (exemplarily shown for the blue molecule) is investigated by time-resolved photoemission using fs extreme ultraviolet (XUV) radiation

Optical excitation across the fundamental band gap between the valence band (in organic semiconductors referred to as highest occupied molecular orbital or HOMO) and the conduction band (lowest unoccupied molecular orbital or LUMO) does not lead to the generation of free charge carriers in the valence and conduction band, but to the formation of new quasiparticles called excitons, i.e., strongly bound electron–hole pairs^18–21^. The energy spectrum of these quasiparticles is characterized by a hydrogenlike series of well-defined states within the HOMO–LUMO band gap as illustrated schematically in Fig. 1a. The unusually large exciton binding energies *E*_Exciton_ in the range of several hundreds of meV up to even 1.0 eV^22–24^ make excitons in organic thin films stable even at elevated sample temperatures. The large value of *E*_Exciton_ is a direct result of the quantum confinement and the correspondingly strong Coulomb interaction between the electron and hole, which are either located on a single molecular site^25^ (Frenkel exciton) or are divided onto two neighboring molecular sites (charge-transfer exciton)^26,27^. Most importantly however, the many-body interactions and the dielectric screening within the organic thin film lead to a significant reduction (renormalization) of the exciton binding energy in molecular films compared to isolated organic molecules^28,29^.

In a similar way, the energy positions of the transport levels of organic thin films, i.e., the HOMO and LUMO level, are also strongly influenced by many-body effects and dielectric screening^30–34^. These energy positions of the transport levels are directly linked to the charge transport properties as well as to the charge injection or extraction barrier between the organic thin films and their environment since they represent the minimum energy for the formation of free charges in an organic material. As schematically depicted in Fig. 1b, the Coulomb repulsion or attraction between added or removed charges and the other charges of a free molecule in the gas phase increases the HOMO–LUMO transport gap $E_{\mathrm{T}}^{{\mathrm{free}}}$ compared to the gap *E*_BG_ of a neutral molecule. In molecular crystals and thin films, however, the transport gap $E_{\mathrm{T}}^{{\mathrm{free}}}$ is again significantly reduced by the dielectric screening^32^. The long-range Coulomb interaction between the injected charge carrier and the polarized molecular environment is associated with a large polarization energy *P* which renormalizes the energy position of the HOMO and LUMO and consequently the transport band gap $E_{\mathrm{T}}^{{\mathrm{bulk}}}$ in molecular films. For this reason, the transport levels HOMO and LUMO can be described as quasiparticles called electronic polarons, i.e., as charged particles that are dressed by the electronic polarizability of the molecular film^31,35^.

Along these lines, it is quite straightforward to expect that the optical and transport properties of organic thin films will be strongly interwoven, as they directly reflect the many-body interactions in organic materials. Experimentally, however, it is a non-trivial challenge to detect their mutual influence, mainly due to the short lifetime of excitons and the lack of temporal resolution in transport measurements that typically detect polaronic effects. To overcome this limitation, we exploit the potential of time-resolved photoelectron spectroscopy (tr-PES) to simultaneously investigate the excited state dynamics as well as the transient changes in the occupied transport levels of a thin film of a prototypical organic semiconductor often employed for organic photovoltaics: the fullerene C_60_. We have grown thin crystalline films of 10 layers of C_60_ on a Ag(111) surface. This film thickness is sufficient to suppress any influence of the Ag(111) substrate on the excited state dynamics of C_60_ and hence allows us to access the intrinsic properties of the organic material^36,37^. A schematic illustration of our experiment is shown in Fig. 1c. First, a femtosecond (fs) optical pump pulse (3.2 eV) excites electrons resonantly across the optical band gap of the organic material leading to the formation of excitonic quasiparticles at local sites within the molecular film, which can be of charge transfer (CT) and Frenkel exciton character^38–40^. Subsequently, a second fs extreme ultraviolet (XUV) pulse (22.2 eV) at a well-defined time delay Δ*t* photo-excites electrons both from the excited states as well as from the occupied part of the band structure into the vacuum, where they are detected by an angle-resolving photoelectron spectrometer^41,42^. The photon energy of the fs-XUV probe pulse is large enough to address a substantial energy range of the excited states and the occupied band structure of C_60_. With respect to the excited states, we are thus able to monitor exciton dynamics, with the possibility to gain insight into the dynamics and charge character of the optically generated excitons, i.e., to distinguish between the formation of the Frenkel and CT excitons in C_60_. Note that this is still a highly intriguing and yet unsolved question for most homomolecular organic semiconductor films, and we will show below that our experiment is capable to address this question. With respect to the dynamics in the occupied band structure of C_60_, we can simultaneously monitor the transient band renormalization and hence gain access to excitation-induced changes of the molecular transport levels. In this way, we are able to determine the correlation between optical and transport properties in organic thin films.

### Excited state dynamics

**Fig. 2:**
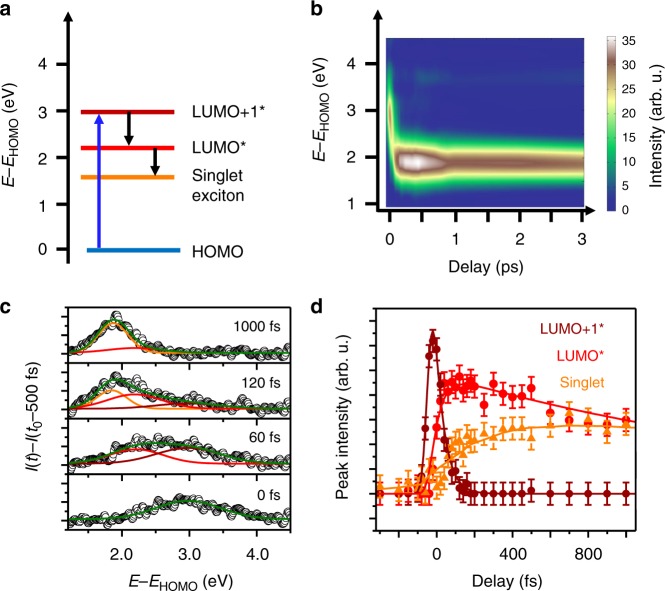
Quasi-particle dynamics of excited excitons in C_60_/Ag(111) (10 ML, *F* = 40 ± 12 µJ cm^−2^). **a** Single particle energy level diagram of the optical quasiparticle states of a C_60_ molecular crystal. The electronic transition induced by the optical excitation is marked with a blue arrow, the subsequent decay steps by black arrows. **b** Two-dimensional *E − E*_HOMO_(*t*) spectrum of the quasiparticle dynamics in the unoccupied states recorded by time-resolved photoemission spectroscopy (tr-PES). Selected energy distribution curves (EDCs) at characteristic time delays are shown in panel **c**. The transient changes in the spectral density can be described by a fitting model considering three excitonic states: the second to lowest unoccupied molecular orbital exciton (LUMO+1*) (*E* − *E*_HOMO_ = 3.0 eV), the LUMO* exciton (*E* − *E*_HOMO_ = 2.2 eV), and the singlet exciton (*E* − *E*_HOMO_ = 1.9 eV). In our experiment, the linewidth of all excited state signals is determined by the time-independent inhomogeneous broadening of the experiment, i.e., by the structural inhomogeneity of the C_6__0_ film as well as by the spectral bandwidth of our laser light source. **d** Temporal intensity evolution of the three excited states shown as brown, red, and orange data points for the LUMO+1*, the LUMO*, and the singlet state. The error bars represent the uncertainty of the fitting parameter in the fitting routine used to analyze the excited state spectra in **c**. The characteristic timescales of the exciton formation and decay are obtained by a dedicated fitting model (solid lines)

We start with the ultrafast quasiparticle dynamics of excitons in a C_60_ crystal. Optical excitation of C_60_ with visible light (3.2 eV) leads to a resonant excitation of excitons, consisting of an electron in the LUMO+1 level and the corresponding hole in the HOMO level. These excitons decay stepwise into energetically lower excitons as illustrated in the energy level diagram in Fig. 2a. This behavior can be directly observed in the two-dimensional plot of the photoemission intensity in Fig. 2b. We find an instantaneous increase of spectral intensity approximately 3 eV above the HOMO level after the optical excitation which rapidly decays to energies of lower lying excited states within the first 100 fs. On longer timescales, the spectral intensity stabilizes at *E* − *E*_HOMO_ = 1.9 eV pointing to a trapping of the excited electron in a state with an exceptionally long lifetime. This trapped state is an intrinsic property of the C_60_ film and not caused by inhomogeneities or domain boundaries of the C_60_ film^43^.

This stepwise decay mechanism can be seen even more clearly in the shape of selected energy distribution curves (EDCs) at characteristic time delays in Fig. 2c, together with our fitting model. At least three states are necessary to model the spectral density for all steps during the exciton decay process^44,45^. The characteristic timescales of the exciton dynamics are illustrated in Fig. 2d. All details of the data analysis procedure are discussed in the section Supplementary Methods. The optical excitation leads to an instantaneous formation of an excitonic state (LUMO+1* exciton, brown curve) which decays within *τ*_LUMO+1,decay_ = 45 ± 10 fs into the next lower lying excitonic level (LUMO* exciton, red curve). A similar fast decay of the LUMO+1* level has also been observed by Shibuta et al. for a C_60_ monolayer on highly oriented pyrolytic graphite^46^. The redistribution of spectral intensity between the LUMO+1* and the LUMO* states in our case occurs on identical timescales (*τ*_LUMO+1*,decay_ = *τ*_LUMO*,rise_) leading to the shift in spectral intensity discussed before. Subsequently, the electron in the LUMO related exciton decays on much longer timescales (τ_LUMO*,decay_ = 2.0 ± 0.3 ps) into a state that is referred to as singlet exciton in literature^37,44,45^ (orange curve) where it becomes trapped for several hundreds of ps. Changing the strength of the optical excitation by changing the laser fluence *F* in the range between *F* = (20 ± 12) and *F* = (60 ± 12) µJ cm^−2^ reveals the same stepwise relaxation process with identical timescales (within the experimental uncertainty). This clearly underlines that the optical excitation strength of our experiment is low enough to investigate the quasiparticle dynamics of optically excited excitons in the so called single-particle limit, i.e., the density of optically excited charge carriers is so low that interactions between neighboring excitons or the formation of trions or bi-excitons do not play a significant role. These observations are fully in line with previous investigations of excitonic lifetimes of organic molecules on various noble metal surfaces obtained by conventional two-photon photoemission experiments where both pump and probe photons have frequencies in the visible range^36,37,44–47^. In contrast, however, using probe pulse photon energies in the XUV range allows us to access the evolution of the occupied molecular levels, i.e., the transport levels and thereby the polaron dynamics in the presence of excitons.

### Transient changes of the occupied valence band structure

**Fig. 3:**
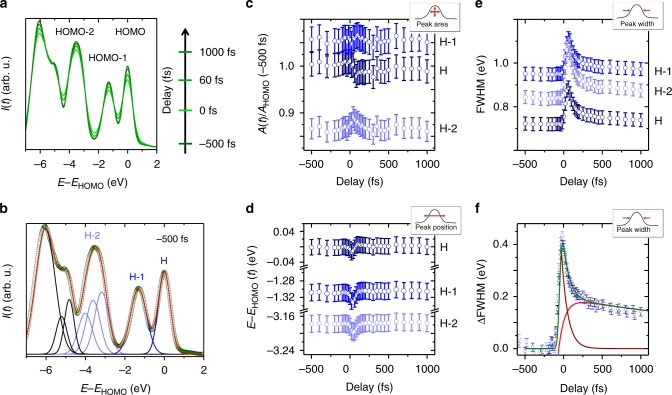
Transient spectral changes of the occupied valence band structure of C_60_/Ag(111). **a** Selected energy distribution curves (EDCs) of the occupied molecular valence band structure (HOMO-X levels) at characteristic time steps. The line shape of these EDCs are analyzed separately at each time step by a dedicated fitting model shown in panel **b**. The corresponding fitting parameters normalized intensity *A*, binding energy position *E − E*_HOMO_, and full width half maximum (FWHM) of each spectroscopic feature are presented in panels **c**–**e**. The error bars represent the uncertainty of the fitting parameter in the fitting routine used to analyze the valence band spectra. **f** Transient broadening (relative change of the FWHM) of each HOMO-X level. These curves are calculated by separating the static broadening of the spectroscopic levels prior to the optical excitation from the optically induced change of the FWHM. The error bars are obtained by propagation of the uncertainties of the original data sets. The solid lines represent a fitting model used to extract the temporal evolution of the transient broadening (ΔFWHM). The data were obtained for a C_60_ coverage of 10 ML and a laser fluence of *F* = 40 ± 12 µJ cm^−2^

Figure 3a shows the general trend of the optically induced band structure dynamics of the occupied molecular levels by displaying selected EDCs for characteristic time steps of the previously observed exciton dynamics in the excited states. First, before optical excitation (*t* = −500 fs), the quasi-static spectral lineshape of the HOMO level as well as the lower lying molecular orbitals (HOMO-X) reflects perfectly the spectroscopic findings of previous studies of C_60_ on various surfaces^20,46,48^. Upon optical excitation and creation of excitons, however, the linewidth of all molecular features increases before transforming back to its original state. The relaxation dynamics is significantly slower than the excitation and occurs only within several hundreds of fs, in analogy to the decay time of the LUMO* exciton. For a more quantitative analysis, the spectra are analyzed by a fitting model (Fig. 3b) adapted from theoretical calculations^48^. A comprehensive discussion of our fitting model can be found in Supplementary Figure 1, the constraints of the fitting procedure are summarized in Supplementary Table 1. The fitting results of the spectral linewidth (full width at half maximum, FWHM), the relative occupation (normalized area A) and the binding energy position of the emission signal of the HOMO, HOMO-1 and HOMO-2 bands are summarized in Fig. 3c–e. We only find a marginal change of the binding energy position during the optical excitation that is identical for all molecular levels. Similarly, the normalized area of all molecular levels remains constant within the experimental uncertainty. The most significant change is a simultaneous increase of approximately 0.4 eV of the spectral linewidth of all molecular features, which is identical for all HOMO bands. This becomes particularly clear when considering the extracted transient broadening of all molecular levels (ΔFWHM) that all collapse onto a single transient ΔFWHM trace in Fig. 3f.

Most interestingly, the transient spectral changes of the HOMO level are identical to those of the other HOMO-X levels, despite the fact that the optical excitation transfers electrons from the HOMO to an excitonic state. This should result in a depopulation of the HOMO and consequently in a lower photoemission yield and possibly in a different broadening of the HOMO feature. However, due to the extremely low number of excitons in the molecular film for our excitation conditions, the optically excited C_60_ molecules do not significantly contribute to the photoemission spectra of the molecular valence band. Instead, the valence band signal is dominated by C_60_ molecules that are not directly affected by the optical excitation, i.e., by molecules mediating the collective dielectric screening of the organic film. Hence, the transient band structure dynamics of the occupied molecular orbitals truly reflects the many-body response of the organic film.

To discuss the temporal evolution of the transient broadening, we will focus on the transient broadening of the HOMO level in Fig. 3f and analyze it by a dedicated fitting model. The initial broadening of the HOMO directly follows the temporal width of the pump pulse pointing to an immediate response of the molecular film to the optical excitation. For the relaxation, i.e., the reduction of the FWHM, we find first a fast decay within *τ*_1_ = 65 ± 20 fs followed by a second step with a time constant of *τ*_2_ = 2.1 ± 0.4 ps. The temporal evolution is qualitatively and even quantitatively almost identical to the excited state dynamics of C_60_ discussed above. Hence, the transient broadening of the valence band spectrum is directly linked to the ultrafast excited state dynamics of the C_60_ film.

These experimental observations can be understood when considering the contributions to the spectral linewidth in organic films. In general, the spectral linewidth depends on two factors: the homogeneous broadening due to the finite lifetime of the photo-hole in the excited state and the inhomogeneous broadening due to structural inhomogeneities in the organic thin films. Homogeneous broadening is usually reflected in a change of the Lorentzian lineshape of the photoemission signal which is inconsistent with our experimental data. Therefore, we propose that the non-interacting excitons in the organic film cause a transient inhomogeneous broadening of the spectroscopic features of the optically non-excited molecules. This hypothesis is fully supported by the following micro-electrostatic model calculations.

### Micro-electrostatic simulations

**Fig. 4:**
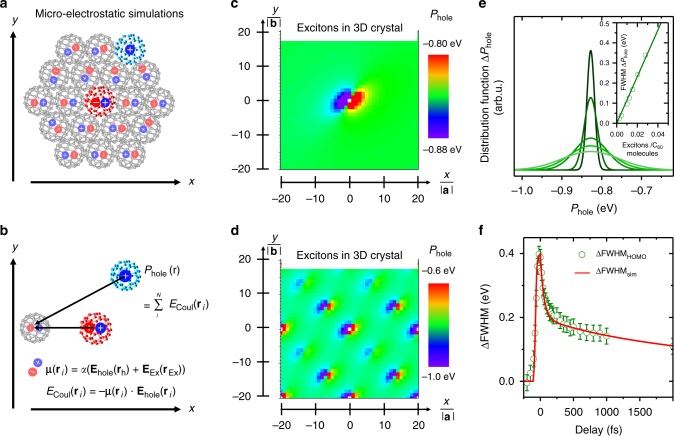
Micro-electrostatic simulations of the energy renormalization of the molecular states. **a** Schematic overview of the micro-electrostatic simulations modeling the transient broadening of the occupied transport level in the presence of an exciton. The exciton (modeled by an electric dipole) creates an electric polarization in the film by inducing dipoles at neighboring sites of highly polarizable C_60_ molecules. These induced dipoles interact with the hole-like transport levels of the film leading to an energy renormalization of the polarization energy *P*_hole_ of the hole-like transport level depending on its relative position in the organic film. Panel **b** illustrates the individual calculation steps of the micro-electrostatic simulations. 2D polarization energy maps *P*_hole_(*x,y*) of the surface layer of a C_60_ face centered cubic (fcc) (111) crystal (slab of 6 layers) are shown for a single exciton (**c**) as well as a regular array of excitons in the first three surface layers with exciton density 0.0025 excitons/C_60_ (**d**). The averaged distribution functions for the polarization energies are shown in panel **e** together with the respective FWHM for various exciton densities as inset. **f** Temporal evaluation of the transient broadening of the polarization energy distribution determined by the micro-electrostatic simulations (red curve, scaled by a factor 1.5). The model considers a stepwise decay between three excited states (LUMO + 1*, LUMO*, and singlet state) of constant dipole strength (*p*_LUMO+1*_ = 1*p_0_ = e*d_C60_, *p*_LUMO*_ = 0.66*p_0_, and *p*_singlet_ = 0.0007*p_0_) and time-dependent population. The density of excited molecules at *t*_0_ was 0.021 excitons/C_60,_ and the temporal evolution of the excited state population was described according to the time-dependent intensity of the excited states in Fig. 2d. More details on the simulations can be found in Supplementary Figure 3. For comparison, the experimentally observed transient broadening of the HOMO level and the corresponding error bars are included as green data points

We have performed the calculations (see Methods) according to refs. ^33,49–51^, where they have been successfully applied to simulate the experimentally observed energy renormalization of the hole-like polaronic transport level (HOMO) of prototypical molecules in molecular thin films. In particular, the energy renormalization of the transport levels of each molecule in an organic film can be estimated by its local polarization energy *P*_hole_(**r**), i.e., by the Coulomb energy between the hole-like transport level on a local molecular site **r** and the induced dipole moments **μ**(**r**_i_) at the other molecular sites **r**_i_. The latter are the result of the high polarizability of organic materials and the corresponding dielectric screening. For quasi-infinite arrays of polarizable molecules, the polarization energy *P*_hole_(**r**) is independent of the position of the hole-carrying molecule in the organic film. This energy defines the global energy renormalization of the HOMO transport level and is hence responsible for the binding energy position of the HOMO levels in photoemission/transport experiments^31–35^. The situation changes significantly in the presence of an excitonic excitation at a local site. This electron–hole pair is expected to coincide with the formation of a transient multipole moment that will also contribute to the strength and direction of the induced dipole moments **μ**(**r**_i_) at each molecular site **r**_i_. In the simplest case, the exciton can be modeled by an electric dipole **p**(**r**_ex_) as shown in Fig. 4a, b (red C_60_ molecule). The local polarization energy *P*_hole_(**r**) can be obtained by the Coulomb energy between the hole-like transport level at **r** (blue C_60_ molecule) with the ensemble of induced dipole moments **μ**(**r**_i_) at the other molecular sites (gray C_60_ molecules). For a single exciton in the surface layer of a C_60_ crystal, the spatial variation of the polarization energy *P*_hole_(**r**) for different molecular sites in the surface layer are shown as 2D polarization energy map in Fig. 4c. Note that only these surface molecular sites are accessible in our tr-PES experiment due to the high surface sensitivity of this technique. We find a clear spatial variation of the polarization energy of a few meV depending on the distance between the non-excited C_60_ molecules (blue C_60_ molecule in Fig. 4a) and the exciton (red C_60_ molecule in Fig. 4a). The energy scale of the spatial variation increases drastically when increasing the density of excitons in the first three layers of the C_60_ film as revealed by the exemplary 2D polarization energy map in Fig. 4d with 0.0025 excitons/C_60_. The corresponding averaged polarization energy distribution functions Δ*P*_hole_ for various exciton densities are shown in Fig. 4e. All Δ*P*_hole_-curves reveal an increasing FWHM in the range of a few 100 meV for increasing exciton densities as illustrated in the inset of Fig. 4e. This behavior qualitatively and even quantitatively reflects the experimentally observed linewidth broadening for various excitation strengths, i.e., different laser fluences. We have analyzed the transient broadening for different fluences in the range between *F* = (20 ± 12) and *F* = (60 ± 12) µJ cm^−^^2^ and found an almost linear increase of the inhomogeneous broadening at *t*_0_ with increasing exciton density in the C_60_ film as shown in Supplementary Figure 2. This clearly demonstrates the severe influence of the optically generated quasi-particles on the energy positions of the transport level of C_60_ molecules in close vicinity of the excited molecules.

Moreover, the temporal evolution of the HOMO and HOMO-X broadening during the exciton decay can also be described by our micro-electrostatic simulations. In accordance with our experimental findings, we modeled the transient population of the excited states by a stepwise decay between LUMO+1*, LUMO*, and so called singlet exciton state. Each excited state is thereby described by an electric dipole **p**(**r**_ex_) with constant dipole strength. The time-dependent population of each excited state follows the dynamics discussed in Fig. 2d. Reducing the strength of the dipole moment **p**(**r**_ex_) of the excitons thereby mimics the decay of the excited state to lower lying excitonic states. A more detailed description of these simulations can be found in Supplementary Figure 3 and Supplementary Note 1.

The best agreement between the experimentally observed transient broadening of the HOMO levels and our simulation is obtained for a relative dipole strength of the LUMO+1* and LUMO* level of *p*_LUMO+1*_/*p*_LUMO*_ = 1.5, and a vanishing dipole strength of the singlet state. The simulated transient broadening ΔFWHM_sim_ curve is shown in Fig. 4f together with the experimentally observed transient broadening of the HOMO level. First of all, the excellent qualitative agreement between our experimental findings and micro-electrostatic simulations provides conclusive evidence that local exciton formation leads to an inhomogeneous broadening of the transport levels in the entire molecular film. Based on the ultrafast fs timescale of the transient inhomogeneous broadening, it must be caused by a purely electronic effect and not by transient structural inhomogeneities, which are usually responsible for the inhomogeneous linewidth of organic films. Hence, we have to conclude that optically excited excitons can act as charge defects in the organic films leading to a strong and instantaneous dielectric response of the entire molecular film. The second important information that can be drawn from our simulations is the qualitative difference in the charge distribution of optically generated excitons in C_60_. In fact, the significant dipole strength of the LUMO+1* and the LUMO* excitons hints to an increased charge separation between the optically generated electron–hole pairs, i.e., to the formation of CT excitons with electrons and holes located on neighboring molecular sites. On the other hand, the vanishing dipole moment of the singlet exciton in our simulation suggests the formation of an electrostatically neutral exciton, i.e., of a Frenkel exciton for which the electron and hole are located on a single molecular site.

## Discussion

Based on our findings, we can propose the following model for the excited state dynamics in C_60_ thin films: optical excitation with 3.2 eV photons results in the formation of LUMO+1* excitons. These excitons exhibit a dominant charge transfer character and the electrons and their corresponding holes are found in the LUMO+1 and HOMO levels of neighboring molecular sites. The existence of such CT excitons in C_60_ at comparable excited state energies has already been predicted theoretically^38–40^. The local charge distribution of these CT excitons instantaneously affects the polarization energy of the surrounding molecules resulting in a transient energy shift of the transport levels. The magnitude and sign of the energy shift depend on the relative position of the molecule and the CT exciton which leads to the transient broadening of all valence states of C_60_ observed in our spatially averaging tr-PES experiment. Within the first 100 fs, the population of the LUMO+1 decays into the LUMO level of the same molecular site and results in the formation of the LUMO* exciton, also with CT character. The different symmetry and spatial distributions of the LUMO+1 and LUMO wave functions cause a reduction of the magnitude of the charge defect leading to a decrease of the transient broadening of the HOMO-X levels of the molecular film. In the subsequent decay step, the LUMO* excitons with dominant CT character transform into singlet excitons with dominant Frenkel-character, i.e., into excitons with the electron and the hole located on the same molecular site. This transition between CT and Frenkel excitons is possible due to the non-vanishing overlap of the molecular wave functions of neighboring C_60_ sites and the (at least for organic materials) relatively large hopping integral in C_60_. At this stage, the transient broadening of the molecular valence levels of the C_60_ film is only determined by the population decay of the LUMO* CT-excitons and not by one of the Frenkel-like singlet excitons. Finally, we would like to point out that the fast timescale of the exciton decay processes points to the absence of any spin-flip scattering process during the exciton decay cascade, i.e., all excitons involved in the decay process have the same singlet spin state^37,44,45^.

In conclusion, we used time-resolved photoemission to investigate the correlation between the optical (exciton) and transport (polaron) properties of organic thin crystals on ultrafast timescales. Focusing on the fullerene C_60_, we find that optical excitation with fs-light results in the formation of non-interacting excitonic states of different charge character which coincides with a transient broadening of the molecular levels of the molecular surrounding. In the subsequent exciton decay process, each energy relaxation step in the excited states is instantaneously reflected in a reduction of the spectral linewidth of all occupied states following precisely the timescale of the excitonic quasi-particle dynamics. The instantaneous spectral changes of the occupied valence band structure are a clear indication of a direct response of the polaronic levels mediated by dielectric screening. This conclusion is further supported by micro-electrostatic simulations modeling the dielectric response of the molecular film to the formation of non-interacting excitons. The excellent agreement between experiment and model simulations clearly highlights the intrinsic correlation between excitons and polarons in organic semiconductor materials and its consequences for all optoelectronic applications. In particular, our results show that optically excited CT excitons in organic materials are pure charge defects that severely modify the transient energy level alignment of the transport levels of the surrounding molecules depending on their distance from the CT exciton. Crucially, these modifications are in the energy range of some hundreds of meV, and will have a profound influence on the performance of organic photovoltaics devices^52^. In this way, our findings can lead to novel concepts of designing the transient energy level alignment of organic molecules in the vicinity of excitons for a more efficient conversion from bound electron–hole pairs (excitons) to free charge carriers.

## Methods

### Sample preparation

The surface of the (111)-oriented silver crystal was cleaned by repeated cycles of argon ion bombardment and subsequent annealing at a temperature of *T*_sample_ = 730 K. The cleanliness of the Ag(111) surface was verified by measuring the surface state at the Γ point of the surface Brillouin zone. Subsequently, the organic material was deposited onto the clean Ag(111) surface at room temperature using a dedicated evaporation source. The success of the sample preparation was confirmed by low energy electron diffraction (LEED) and photoelectron spectroscopy of the molecular valence band. The appearance of a LEED pattern with narrow diffraction spots as well as of a small linewidth of the molecular emission feature in the static photoemission signal suggests a long-range crystalline order with a low defect density. The molecular coverage was controlled by the evaporation time which was calibrated to the evaporation time of a C_60_ monolayer film on Ag(111). The latter was identified by its characteristic LEED pattern which changes for higher molecular coverage.

### Time-resolved photoelectron spectroscopy

The photoemission experiments were conducted under ultrahigh vacuum conditions with a base pressure better than 5 × 10^−10^ mbar. Photoelectrons were detected with a hemispherical electron spectrometer (SPECS Phoibos 150) and a 2D detection system. All photoemission data were acquired in normal emission geometry and with an angle of incidence of the pump and probe pulse of 45° with respect to the sample normal. The femtosecond extreme ultraviolet (fs-XUV) radiation (22.2 eV, p-polarized) was obtained by high harmonic generation (HHG) using the second harmonic (390 nm) of a titanium sapphire laser amplifier system (repetition rate 10 kHz, pulse duration < 40 fs) to drive the HHG process^41^. The optical excitation of the organic material was also performed with the second harmonic of the amplifier system (3.17 ± 0.04 eV, bandwidth 80 meV, p-polarized). A spectrum of the pump pulse is shown in Supplementary Figure 4. The pulse durations of the optical pump and fs-XUV probe were determined to be 58 ± 5 and 40 ± 5 fs, respectively, the cross-correlation of pump- and probe at the position of the sample was 70 fs, both obtained using a 1.58 eV pump pulse. Prior to each time-resolved experiment, the spatial overlap between the pump and the probe pulse was optimized using a fluorescent plate which was placed on the manipulator at the focus position of the analyzer. The spatial overlap was actively stabilized during the experiment to correct for spatial drift of the pump and probe beams. This is achieved by constantly monitoring the beam position of the fundamental laser beam at two well-defined positions in the laser beamline using two CCD cameras. Any lateral draft of the laser beam is compensated by two motorized mirrors installed in the beamline. The beam size of the pump beam on the sample surface was determined to be (250 ± 6) μm × (415 ± 8) μm, the one of the XUV-probe beam (250 ± 6) μm × (300 ± 6) μm. The larger diameter of the pump spot ensures a homogeneous optical excitation of the probed area of the C_60_ film. During the tr-PES experiments, we carefully monitored the radiation-induced degradation of the sample. Only marginal changes of the lineshape of the C_60_ valence bands were observed even after several hours exposure with fs-XUV and fs-optical radiation in the visible range. Despite this high stability of the organic film, we regularly changed the sample position to avoid radiation-induced damage of the sample. Additional information regarding the data analysis procedure can be found in Supplementary Methods.

The analysis of the photoemission data was performed with a least square fit using Gaussian functions, the uncertainties of the fitting results were estimated by the maximum uncertainty of a Monte Carlo error analysis implemented in CASAXPS. More details about the data analysis procedure can be found in Supplementary Figure 1.

### Micro-electrostatic simulations

The micro-electrostatic calculations were performed for an fcc(111) crystal of C_60_ molecules arranged in a slab with nine molecular layers, each layer consisting of a grid of 340 × 340 lattice points, i.e., 340 × 340 C_60_ molecules. Each C_60_ molecule was treated as a point object with polarizability *α*, but without intrinsic molecular dipole moment. The local polarization energy *P*_hole_(**r**) was evaluated for each C_60_ molecule on a 40 × 40 grid centered in the surface layer of the C_60_ slab. The polarization energy *P*_hole_(**r**) is calculated for each position on this sub-lattice by the total Coulomb energy between the electric field of the point charge **E**_PC_(**r**_*i*_) (representing the photo-hole, i.e., the polaron in the organic film) and the induced dipole moments at each molecular site **r**_*i*_ of the entire C_60_ slab:

1 $$P_{{\mathrm{hole}}}\left( {\mathbf{r}} \right) = - \mathop {\sum }\limits_i {\mathbf{\mu}}\left( {{\mathbf{r}}_{i}} \right) \cdot {\mathbf{E}}_{\mathrm{PC}}\left( {{\mathbf{r}}_{i} - {\mathbf{r}}} \right) = - \mathop {\sum }\limits_i {\mathbf{\mu }}\left( {{\mathbf{r}}_{i}} \right) \cdot \frac{{e\left( {{\mathbf{r}}_{i} - {\mathbf{r}}} \right)}}{{4{\mathrm{\pi }}\varepsilon _0\varepsilon |{\mathbf{r}}_{i} - {\mathbf{r}}|^3}}$$

The dielectric constant *ε* = 4.4 was taken from literature^53^. The induced dipole moment **μ**(**r**_*i*_) at each site **r**_*i*_ in the C_60_ slab is obtained by

2 $${\mathbf{\mu }}\left( {{\mathbf{r}}_i} \right) = \alpha \;{\mathbf{E}}_{{\mathrm{total}}}({\mathbf{r}}_i) \cdot$$

Due to the high symmetry of C_60_ we employed an isotropic polarizability constant of *α *= 90 Å^3^$\varepsilon _{0}.$^35^ The total electric field **E**_total_(**r**_*i*_)

3 $${\mathbf{E}}_{{\mathrm{total}}}\left( {{\mathbf{r}}_i} \right) = \frac{1}{{4{\mathrm{\pi }}\varepsilon _0\varepsilon }}\left( {\frac{{{\mathbf{r}}_i - {\mathbf{r}}}}{{|{\mathbf{r}}_i - {\mathbf{r}}|^3}} + \mathop {\sum }\limits_{{j}} \frac{{3\;{\mathbf{r}}_i{\mathbf{r}}_j{\mathbf{p}}( {{\mathbf{r}}_j} ){\mathrm{/}}| {{\mathbf{r}}_j} |^2 - {\mathbf{p}}({\mathbf{r}}_j)}}{{|{\mathbf{r}}_j|^3}}} \right)$$

consists of the electrostatic field of the point charge (first term in the bracket above) at **r** and of the electrostatic field created by the *j* excitons in the molecular film located at sites **r**_*j*_ (second term in the bracket above). The electric field of each exciton is approximated by an electrostatic dipole **p**(**r**_*j*_) oriented along the *x*-direction of the C_60_ slab. The strength of the dipole is modeled by |*ed*_C60_| with *d*_C60_ = 10 Å. This value corresponds to the distance between two lattice sites and hence to the upper limit of spatial expansion of the excitonic wave function of C_60_.

## Supplementary information

Supplementary Information

## Data Availability

The data sets obtained and analyzed during the current study are available from the corresponding author on reasonable request.

## Code availability

The micro-electrostatic model calculations were performed with a new dedicated code implemented in the programming language C++. The C++ code is available from the corresponding author on reasonable request.
