# Supplementary material for: Strong modification of the transport level alignment in organic materials after optical excitation
Source: Nat Commun. 2019 Apr 1;10:1470. doi: 10.1038/s41467-019-09136-7 (PMC6443800; doi:10.1038/s41467-019-09136-7)
Supplement: Supplementary file 1 — Supplementary Information [file 41467_2019_9136_MOESM1_ESM.pdf]

Strong modification of the transport level alignment in organic materials after optical excitation

by

B. Stadtmüller et al.

Supplementary Figures

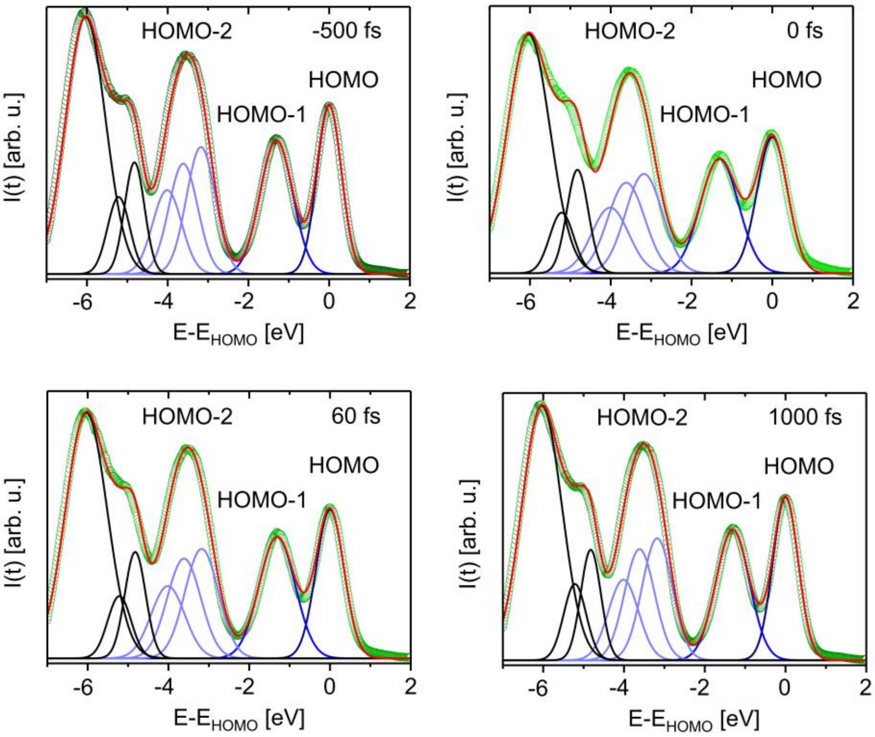

Supplementary Figure 1. Data analysis procedure of the time-resolved photoemission data. The data acquisition of the photoemission yield was performed in the snapshot mode of our photoemission analyser system using a pass energy in the range of 75 to 100eV. The time-dependent changes of the photoemission signal were obtained by recording a 2D photoemission image (energy vs. emission angle) for each time delay between the optical pump and XUV-probe pulse. These so called delay scans were repeated multiple times to improve the signal to noise ratio of the experimental data set. The 1D photoemission spectra of the occupied valence band region were extracted for each time delay from a small angle range (3°) around the Γ-point of the C_{60} surface Brillouin zone. The data analysis of these 1D spectra was performed separately for each spectrum of each delay scan in two subsequent steps. First, we subtracted the secondary electron background from each spectrum using a single exponential decay function with fixed parameters. To avoid artefacts in the subsequent fitting procedure, the parameters for the background subtraction were kept constant for every spectra of a single delay scan. Exemplary spectra after background subtraction are shown here for four selected time delays. Subsequently, we performed a spectral analysis to quantify the relative changes in peak area A, peak position E_B and peak width (FWHM) for each spectroscopic feature by using a dedicated fitting model. The latter was based on density functional theory calculations of a free C_{60} molecule [1] and were adapted to describe our photoemission data with the least amount of Gaussian functions possible. The HOMO and HOMO-1 levels only consist of one π-band and can each be modelled by a single Gaussian function. In contrast, the HOMO-2 and HOMO-3 level reveal a superposition of several molecular orbitals which overlap in energy. In order to obtain a reliable fitting result, we constrained the relative peak position, relative area, and the relative width of the three Gaussian functions describing the HOMO-2 and HOMO-3, respectively. The constraints used in our fitting model were optimized by fitting the photoemission data at *t*=-500fs and are summarized in the supplementary table 1. In this way, we obtain three fitting parameters for each molecular feature: the area (A), the peak position and the width (FHWM).

Exemplary fitting results for four characteristic time delays are shown here. The experimental data are shown as points, the envelope of our fitting model as red curve. The entire fitting model is plotted as coloured lines underneath the experimental data. The fitting analysis yields one energy position, one width and one area for every molecular orbital and each time step of a single delay scan. The fitting results of this data analysis procedure are shown in fig. 3c,d,e of the main text.

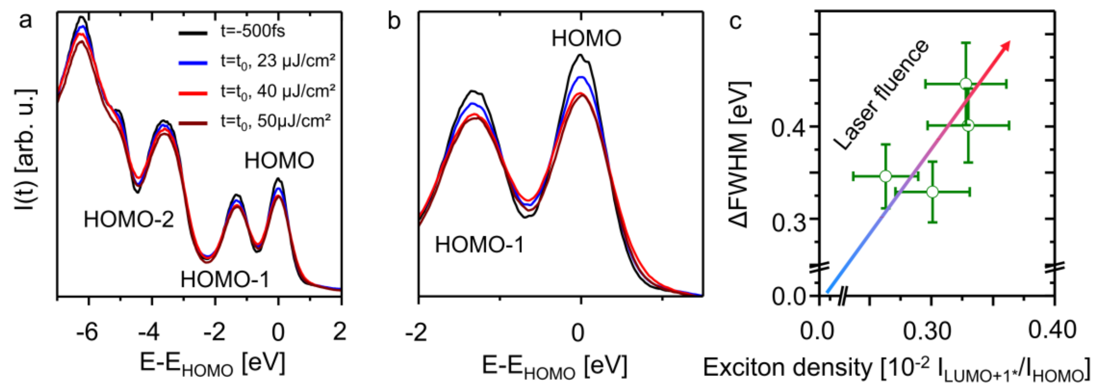

Supplementary Figure 2. Time-resolved photoemission data recorded at different laser fluences. (a) Time-resolved photoemission data recorded at t_0 for three different fluences: (23±12) µJ cm^{-2} (blue curve), (40±12) µJ cm^{-2} (red curve), and (50±12) µJ cm^{-2} (brown curve). The data shown here are raw data without background subtraction. A valence band spectra of the undisturbed C_{60} film recorded before t_0 is included as black curve for comparison. A detailed view onto the transient changes of the HOMO and HOMO-1 features are shown in panel b. The experimentally obtained relation between the density of exited excitons and the transient broadening of the occupied molecular levels is shown in panel c. The exciton density upon the optical excitation was determined from the intensity of the LUMO+1* level at t_0 normalized to the intensity of the HOMO level prior to the optical excitation. As discussed in the main text of the manuscript, the transient broadening of all molecules levels increases with increasing laser fluence.

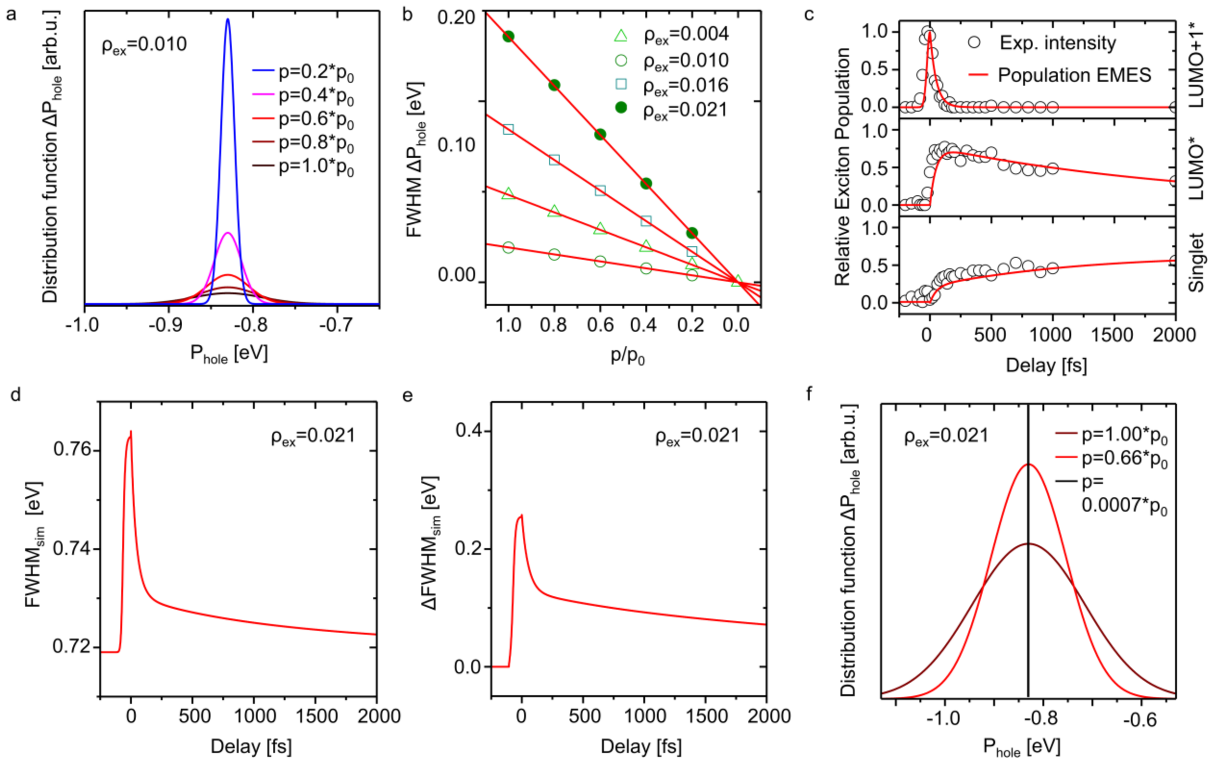

Supplementary Figure 3. Time-resolved micro-electrostatic simulations of the polarization energy. (a) Distribution functions ΔP_{hole} of the polarization energies P_{hole} calculated for different electrostatic dipole moments p. The dipole moments p model the optically excited excitons in the C_{60} film. The distribution functions ΔP_{hole} were extracted from 2D polarization energy maps $P_{\mathrm{hole}}(x,y)$ of the surface layer of a C_{60} face center cubic (fcc) (111) crystal (slap of 6 layers). These maps were calculated for a regular array of excitons located in the first three surface layers with changing electrostatic dipole moments **p** and a constant exciton density 0.010 excitons/C_{60}. The distribution function ΔP_{hole} obtained for the electrostatic dipole **p**=$\mathbf{p_0}$ corresponds to the polarization energy distribution function ΔP_{hole} at t_0, i.e., right after the optical excitation, in analogy to the distribution functions shown in Fig. 4 of the main manuscript (see also Method section of main manuscript). The decay process of the excitons in the molecular film was described by a reduction of the strength of the dipole **p**. It is clearly visible in panel supplementary figure 3(a) that decreasing the strength of the dipole moment **p** results in a continuous reduction of the FWHM of the distribution functions ΔP_{hole}. but not in an energy shift of the distribution function. The FWHM decreases linear with decreasing strength of the dipole moment for all exciton densities as shown in panel (b) and becomes zero as soon as the dipole moments vanish, i.e., **p**=0. Panels (c)-(f) illustrate our approach to determine the temporal evolution of the transient linewidth broadening of all molecular levels upon optical excitation in the framework of the extended micro-electrostatic simulation. For a more detailed description of our calculations, please refer to the supplementary note 1.

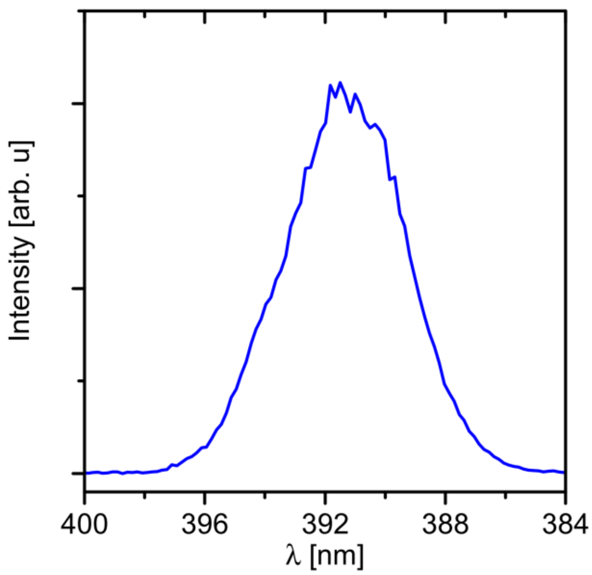

Supplementary Figure 4. Pump spectrum The pump beam spectrum after second harmonic generation has been measured using a spectrometer. The center of mass of this spectral distribution is $\lambda_c = 391.3$ nm, corresponding to $E_{\mathrm{ph,pump}} = 3.17$ eV and the width is $\Delta\lambda_{\mathrm{FWHM}} = 4.8$ nm, corresponding to $\Delta E_{\mathrm{ph,pump,FWHM}} = 0.04$ eV.

Supplementary Tables

Spectroscopic Feature	Area A	E_B [eV]	FWHM
HOMO	A_{HOMO}	E_{HOMO}	$FWHM_{HOMO}$
HOMO-1	A_{HOMO-1}	E_{HOMO-1}	$FWHM_{HOMO-1}$
HOMO-2, Peak 1	A_{HOMO-2}	E_{HOMO-2}	$FWHM_{HOMO-2}$
HOMO-2, Peak 2	$0.91*A_{HOMO-2}$	$E_{HOMO-2}-0.85$	$1.00*FWHM_{HOMO-2}$
HOMO-2, Peak 3	$0.66*A_{HOMO-2}$	$E_{HOMO-2}-1.63$	$1.00*FWHM_{HOMO-2}$
HOMO-3, Peak 1	A_{HOMO-3}	E_{HOMO-3}	$FWHM_{HOMO-3}$
HOMO-3, Peak 2	$0.65*A_{HOMO-3}$	$E_{HOMO-3}-0.77$	$1.12*FWHM_{HOMO-3}$
HOMO-3, Peak 3	$4.61*A_{HOMO-3}$	$E_{HOMO-3}-2.37$	$2.00*FWHM_{HOMO-3}$

Supplementary Table 1. Parameter and constrains of our fitting model for the valence states. Binding energies E_B, peak widths FHWM, and relative intensities (area A) for all peaks of the fitting model shown in supplementary figure 1 as well as in Fig 3b of the main text. The parameters of the three Gaussian peaks assigned to the HOMO-2 and HOMO-3 are constrained to the parameters of the peak at the lowest binding energy (Peak 1).

Supplementary Notes

Supplementary Note 1

To describe the time-dependent broadening, we have selected three discrete dipole moments **p** and one exciton density ρ_{ex} to model the charge defects caused by the LUMO+1*, the LUMO* as well as the singlet exciton level. For each dipole strength **p**, we calculated the distribution function ΔP_{hole} of the polarization energy (inhomogeneous broadening) using our micro-electrostatic simulations (see also Method section of main manuscript). The overall broadening of the distribution function in the presence of three different types of excitons is calculated by a linear superposition of the three distribution function ΔP_{hole} for three dipoles (LUMO+1*, LUMO*, and singlet) multiplied by their relative occupation $f_i(t)$:

$$\Delta P_{\text{hole}}^{\text{total}}(E,t) = f_{\text{LUMO+1*}}(t)\,\Delta P_{\text{hole}}^{\text{LUMO+1*}}(E) + f_{\text{LUMO+1*}}(t)\,\Delta P_{\text{hole}}^{\text{LUMO*}}(E) + f_{\text{Singlet}}(t)\,\Delta P_{\text{hole}}^{\text{Singlet}}(E) \qquad 4$$

This results in series of distribution functions for each time delay. The relative occupation $f_i(t)$ of the excited states is modelled by a stepwise decay process of the excited states observed in our tr-PES experiment. The temporal evolution of $f_i(t)$ is shown in panel c together with the normalized intensity distribution of the excited states extracted from our tr-PES data, see also Fig. 2d of the main text.
For a realistic description of our experimentally observed transient broadening $\Delta FWHM(t)$ discussed in the main text, each distribution function $\Delta P_{\text{hole}}^{\text{total}}(E,t)$ was convoluted with a Gaussian function $G(\sigma,E)$ at each time delay to consider the static inhomogeneous broadening of the molecular levels due to structural inhomogeneities of the molecular film:

$$\Delta FWHM_{\text{hole}}^{\text{total}}(E,t) = \Delta P_{\text{hole}}^{\text{total}}(E,t) \otimes G(FWHM_{\text{static}}, E). \qquad 5$$

The simulated transient broadening $\Delta FWHM_{sim}(t)$ was subsequently extracted in two steps, in analogy to the analysis of the tr-PES data. First, each individual distribution function $\Delta FWHM_{\text{hole}}^{\text{total}}(E,t)$ is fitted with a Gaussian function for all time delay to obtain the transient linewidth $FWHM_{sim}(t)$ of the simulated distribution function. Subsequently, the transient broadening $\Delta FWHM_{sim}(t)$ is calculated by

$$\Delta FWHM_{\text{sim}}(t) = \sqrt{FWHM_{\text{sim}}(t)^2 - FWHM_{\text{static}}{}^2}. \qquad 6$$

The simulated transient linewidth $FWHM_{sim}(t)$ shown in panel d and the transient broadening $\Delta FWHM_{sim}(t)$ shown in panel e were obtained for dipole strengths of $p_{LUMO+1*} = p_0 = ed_{\mathbf{C60}}$, $p_{LUMO*} = 0.66p_0$, and $p_{singlet} = 0.0007p_0$. The density of the optically excited LUMO+1* levels was 0.021 excitons/C_{60} in the simulations. The corresponding distribution functions $\Delta P_{hole}(E)$ for these parameters are shown in panel f. In Fig. 4f of the manuscript, the simulated transient broadening $\Delta FWHM_{sim}(t)$ is shown together with the experimentally obtained transient broadening of the HOMO level. Note that the simulated curve was scaled by factor 1.5 to quantitatively match the experimental findings. Such small quantitative differences are frequently observed when comparing experimental data with micro-electrostatic simulations due to the simplicity of the model [2]. However, the overall shape of the simulated transient broadening $\Delta FWHM_{sim}(t)$ can perfectly describe the temporal evolution of the experimental data and hence can qualitatively explain the observed phenomena.
The excellent qualitative agreement between our micro-electrostatic simulations and our experimental findings allow us to gain insight into the charge character of the exited states in C_{60}. The

parameters of our simulations strongly suggest that the excited level LUMO+1* and the LUMO* exhibit a significant (microscopic) charge distribution and can hence modify the energies of the transport levels of the surrounding molecules. Such a charge distribution is characteristic for excitons with dominant charge transfer exciton character. In contrast, the vanishing dipole moment of the singlet level in our simulations indicates that the corresponding singlet exciton does not reveal a non-vanishing charge distribution. This is consistent with an exciton of dominant Frenkel exciton character.

Supplementary Methods

Details on the Spectral Analysis of Excited States

To extract the transient occupation of the different excited states from the raw data acquired in the angle-resolved mode of our photoemission spectrometer, we generated energy distribution curves (EDCs) for each time delay by integrating over the angular information obtained in our experiment (14° acceptance angle) and by averaging these 1D spectra over all delay scans recorded for one pump laser fluence. For the 1D spectra, an exponential background was subtracted for every step in the time delay scan. The data obtained this way is plotted in Fig. 2b and c for distinct time steps. Subsequently, we analysed the spectral shape of the exited states by fitting each spectrum individually with three Gaussian curves. During the fitting procedure, the peak width (FWHM) and the peak positions were constrained to constant values. The peak positions were taken from [3], the FWHM was optimized by iteratively repeating the fitting procedure with different, but time-independent FWHM values. The only free fitting parameter is the peak intensity. The best fitting results are shown in Fig. 2c for selected time steps, the transient intensity of all three excited states in Fig. 2d.

Details on the Analysis of the Population Decay constant of the excited states

The population decay constants of the excited states LUMO+1* and LUMO* were obtained by fitting their time dependent intensity evolution by the exponential fitting function $F_{LUMO+1^*}(t)$ and $F_{LUMO^*}(t)$:

$$F_{\mathrm{LUMO+1^*}}(t) = \begin{cases} I_0 & t < t_0 \\ I_1\, \mathrm{e}^{-(t-t_0)/\tau_{\mathrm{LUMO+1^*}}} & t \geq t_0 \end{cases} \qquad 7$$

$$F_{\mathrm{LUMO^*}}(t) = \begin{cases} I_0 & t < t_0 \\ I_2\, \mathrm{e}^{-(t-t_0)/\tau_{\mathrm{LUMO^*}}} \left(1 - \mathrm{e}^{-(t-t_0)/\tau_{\mathrm{LUMO\_R}}}\right) & t \geq t_0 \end{cases} \qquad 8$$

I_0 is the background intensity, τ_{LUMO+1^*} the decay constant of the LUMO+1* level, τ_{LUMO_R} the rise time of the intensity of the LUMO* level, and τ_{LUMO} the decay constant of the LUMO* level. For the data analysis, the fit functions were convoluted with a normalized Gaussian function $G(\Delta\tau_{FWHM},t)$ with $\Delta\tau_{FWHM}$ = 70 fs. The latter value corresponds to the crosscorrelation of pump and probe on the sample surface and was determined experimentally.
The best fitting results were obtained for $\tau_{LUMO\_R} = \tau_{LUMO+1^*}$. The corresponding fitting functions are shown as solid lines in Fig. 2d of the main manuscript.

Details on the Analysis of the Transient Broadening of the Occupied Valence Band States

The time constants of the temporal evolution of the transient broadening of the occupied valence levels was obtained by a very similar fitting procedure as discussed above for the excited states. The transient linewidth is modelled by the fit function $F_B(t)$

$$I(t) = \begin{cases} I_0 & t < t_0 \\ I_0 + I_1\,\mathrm{e}^{-(t-t_0)/\tau_{\mathrm{LUMO+1^*}}} + I_2\,\mathrm{e}^{-(t-t_0)/\tau_{\mathrm{LUMO^*}}}\left(1 - \mathrm{e}^{-(t-t_0)/\tau_{\mathrm{LUMO\_R}}}\right) & t \geq t_0 \end{cases} \quad 9$$

which has been convoluted with a Gaussian function with $\Delta\tau_{FWHM}$ = 70 fs to consider the temporal broadening of the pump and probe pulse (crosscorrelation of pump and probe). The fit function describes a double exponential decay process to model the two subsequent decay steps observed in the excited states. Again, the best fitting results were obtained for $\tau_{LUMO\_R} = \tau_{LUMO+1^*}$. The best fitting result is shown in Fig. 3f of the main text.

Supplementary References

1. J. H. Weaver et al., *Electronic structure of solid C60: Experiment and theory*, Phys Rev Lett 66, 1741 (1991)
2. K. A. Cochrane, A. Schiffrin, T. S. Roussy, M. Capsoni and S. A. Burke, Pronounced polarization-induced energy level shifts at boundaries of organic semiconductor nanostructures, Nat Commun 6, 8312 (2015)
3. R. Jacquemin, S. Kraus, W. Eberhardt, Direct observation of the dynamics of excited electronic states in solids, Solid State Commun 105, 449–453 (1998)
